# The effect of Baduanjin on body mass and body composition of college students: A randomized controlled trial

**DOI:** 10.1097/MD.0000000000040393

**Published:** 2024-11-01

**Authors:** Nana Wen, Fang Zhao, Shanshan Sun, Jian Xiong, Guohua Zheng

**Affiliations:** a College of Nursing and Health Management, Shanghai University of Medicine and Health Sciences, Shanghai City, China; b Department of Graduate School, Shanghai University of Traditional Chinese Medicine, Shanghai City, China.

**Keywords:** Baduanjin, body composition, college student, randomized controlled trial

## Abstract

**Background:**

Previous studies have also found that Baduanjin training can improve health-related physical fitness in young adults. However, it is unclear whether Baduanjin training can improve body mass and body composition in young adults. The aim of this study was to investigate the effects of the Baduanjin training on body mass and body composition in college students.

**Methods:**

This was a single-center, randomized controlled trial comparing 12 weeks of Baduanjin exercise training group (BEG) at a frequency of 60 minutes per day, 4 days per week with no special exercise control group (CG) on the health-related physical fitness in college students. Body mass and body composition were assessed using the body mass index, waist-to-hip ratio, and Inbody 720 devices. A total of 93 college students (56 in the BEG and 37 in the CG) completed 2 body composition assessments and were included in the analysis. A mixed linear model was used to analyze the effect of the Baduanjin exercise intervention.

**Results:**

After 12 weeks of intervention, the body mass index and waist-to-hip ratio in the BEG were significantly lower than that in the CG (*P* =.007 and *P* =.028) with a moderate effect size (Cohen *d* = 0.584 and 0.474) and a significant interaction effect of group by time (*P* =.007 and.028). The fat mass indicators of body composition including percent body fat, body fat mass, and body fat mass of both arms, both legs and trunk in the BEG were significantly lower than those in the CG (all *P* <.05), with a moderate effect size (Cohen *d* from 0.452 to 0.624) and a significant interaction effect of group by time (all *P* <.05); no significant differences were found in the total body composition indicators and the fat-free mass indicators of body composition between the 2 groups.

**Conclusion:**

Regular Baduanjin exercise training may be beneficial for improving body composition in young adults.

## 1. Introduction

The college years are a critical transition from adolescence to adulthood, and is an important period for establishing behaviors that affect long-term health and chronic disease risk. Therefore, college students have a responsibility for their own health care.^[1]^ However, many college students engage in behaviors or lifestyles, such as physical inactivity or unhealthy eating habits, that reduce the likelihood of optimal health and increase the likelihood of overweight and obesity.^[2,3]^ Studies have shown that approximately 30% of college students are overweight or obese, and 75% of students experience a significant weight change during their college years.^[4,5]^ Cohort studies have shown that excess body mass in young adulthood is likely to be associated with a high risk of cardiometabolic or other chronic diseases, even premature death, and that body mass index (BMI) has a stronger predictive value for mortality at younger ages than at older ages;^[6,7]^ the possible reason being that those who maintain a higher weight in early adulthood suffer more adverse metabolic effects of being overweight than those who become heavy later in life,^[8]^ suggesting the need for additional health promotion strategies on college campuses and for universities need to recognize their role in promoting healthy weight maintenance.

Physical activity is important for the overall well-being of college students, and regular physical activity not only benefits the physical health, but also has a positive impact on their academic career and their psychological and social development throughout their college years.^[9]^ However, physical activity levels among college students remain alarmingly low, and their participation in physical activity has declined significantly while sedentary behavior has increased.^[10]^ A scoping review of physical activity among college students found that only 60% of students met moderate physical activity recommendations,^[11]^ and the barriers to participation were identified as the interpersonal, intrapersonal, or structural factors such as lack of motivation, lack of friends, peer influence, homework, class schedule, and lack of accessible places.^[12,13]^ Therefore, interventions or programs to promote physical activity should focus on noncompetitive exercises that students can do on their own time.^[14]^ Baduanjin exercise is a type of traditional Chinese qigong exercise that originated in ancient China and integrates body and mind, consisting of 8 independent, gentle, and slow movements and postures with low-to-moderate exercise intensity.^[15]^ Baduanjin exercise emphasizes physical and mental concentration, the coordination between symmetrical posture and movement, and harmony between the breathing and meditation not only excavating meridians, but also helping to improve *yin* and *yang* balance and *qi*-*blood* circulation.^[16,17]^ Compared with the conventional competitive sports, Baduanjin exercise could be practiced individually, without the constraints of time, place and partner cooperation, and is therefore more conducive to exercise compliance. Studies have shown that regular Baduanjin exercise training could improve physical functions such as muscle strength, balance, and cardiorespiratory fitness in the middle-aged and elderly people.^[18,19]^ A growing number of studies are also focusing on the benefits of Baduanjin training on the physical and mental health of young people, such as college students.^[20–23]^ We recently completed a randomized controlled trial (RCT) comparing regular Baduanjin exercise training with no specific exercise intervention on the health-related physical fitness in college students.^[24]^ The aim of this analysis was to investigate the effect of 12 weeks Baduanjin exercise training on body mass and body composition of college students based on the data of the previous RCT.

## 2. Methods

### 2.1. Subjects and study design

**Figure 1.**
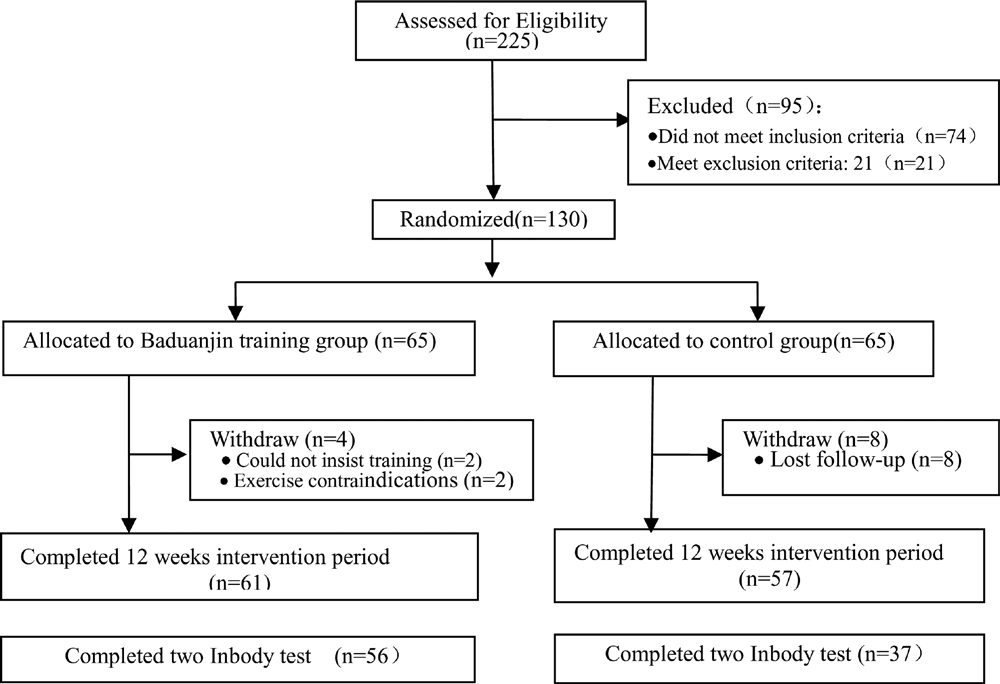
Participants flow diagram.

This RCT was conducted on the campus of SUMHS from May 2018 to October 2019. The original study was registered with the Chinese Trial Registry (ChiCTR-IOR-17013011, http://www.chictr.ogr.cn). The protocol of this study has been reported previously.^[25]^ Briefly, the eligible college students were recruited and randomly allocated to the Baduanjin exercise training group (BEG) or no specific exercise intervention control group (CG). The sample size was calculated based on a predicted 5% improvement in health-related physical fitness scores between the 2 groups after the intervention, with 80% power (*α* = 0.05) and a maximum expected dropout rate of 20%. Due to attrition and loss of data, the final sample consisted of 93 participants (Fig. 1). This study was conducted in accordance with the Declaration of Helsinki and approved by the Ethics Committee of SUMHS (approval number: 2017ZGH). All participants provided written informed consent prior to participation.

### 2.2. Intervention protocol

The detailed protocol was described elsewhere.^[25]^ Briefly, participants in the Baduanjin exercise group received 12 weeks of supervised Baduanjin training at the university gymnasium at a frequency of 60 minutes per day, 4 days per week, and 2 qualified coaches were employed to teach the Baduanjin training. Participants in the CG were informed to maintain their original lifestyle during the 12-week intervention period. All participants in both groups were asked to record their daily physical activity using a pedometer in their mobile phones during the intervention period.

### 2.3. Body mass and body composition assessment

Body mass was assessed by the BMI and the waist to hip ratio (WHR), and BMI and WHR were calculated using the formula (BMI = weight (kilogram, kg)/height (meters, m^2^); WHR = waist circumference (cm)/hip circumference (cm)), in which the waist and hip circumferences, and body height and weight were measured using the weight measuring device manufactured by Zhongtitongfang Co., Ltd., Beijing (product type ZTTF-CSTF-ST). Body composition was measured using the Inbody 720 device (Biospace Co.), which consists of 14 main indicators, including total body composition indicators (basal metabolic rate [BMR], total body water [TBW], total protein and minerals, fat mass indicators of body composition (body fat mass [BFM], percent body fat [PBF], right arm BFM, left arm BFM, right leg BFM, left leg BFM, trunk BFM) and fat-free mass (FFM) indicators of body composition (FFM, soft lean mass [SLM], skeletal muscle mass [SMM]). Body mass and body composition data were collected at the baseline and after the 12-week intervention period by the physical education teachers who were not involved in this study.

### 2.4. Statistical analysis

Continuous variables were expressed as mean (standard deviation/standard error) or median (interquartile range), whereas categorical variables were expressed as numbers (percentages). Baseline characteristics between 2 groups were compared using the two-sample independent *t* test/Mann–Whitney test for the continuous data and the Pearson *χ*^2^ test for categorical data. The differences in body mass and body composition indicators between the 2 groups at baseline and after the 12-week intervention were analyzed using the 2 independent samples *t* test/ Mann–Whitney test, and the effect size between the 2 groups after the intervention was calculated using Cohen d values, where 0.2, 0.5 and 0.8 were considered small, medium, and large effects, respectively.^[26]^ The mixed linear model with restricted maximum likelihood was used to analyze the interaction effect of group by time. Missing data were imputed using a multiple imputation method. All analyses were performed using SPSS 21.0 software (IBM, Chicago, IL), and a *P*-value of <.05 was considered statistically significant.

## 3. Results

### 3.1. Comparison of baseline characteristics between groups

A total of 93 participants (56 participants in the BEG and 37 participants in the CG) completed 2 Inbody examinations. Comparison of the baseline demographic characteristics, body mass and body composition indicators between the 2 groups is summarized in Table 1. There were no differences between the 2 groups at baseline.

**Table 1:** Comparison of the baseline demographic characteristics, body mass, and body composition indictors between the 2 groups.

	BEG (mean ± SD) n = 56	CG (mean ± SD) n = 37	t/*χ*^2^	*P*-value
Basic demographic characteristics of participants				
Age (years)	19.66 ± 0.79	19.49 ± 0.99	0.939	.350
Gender (male/female, n)	4/56	2/37	-0.171	.865
Weight (kg)	56.46 ± 9.03	56.60 ± 10.28	-0.072	.943
Body mass				
BMI (kg/m^2^)	21.53 ± 3.01	21.34 ± 3.32	0.296	.768
WHR (%)	0.80 ± 0.04	0.81 ± 0.05	-0.887	.378
Overall indicator of BC				
BMR (kcal/d)	1246.68 ± 128.82	1256.51 ± 131.64	-0.357	.722
TBW (kg)	29.70 ± 4.37	30.04 ± 4.43	-0.364	.717
Total protein (kg)	7.90 ± 1.20	7.99 ± 1.23	-0.361	.719
Total minerals (kg)	2.97 ± 0.41	3.00 ± 0.45	-0.277	.783
Fat mass indicators of BC				
PBF (%)	27.61 ± 6.70	26.80 ± 6.99	0.561	.576
BFM (kg)	15.87 ± 5.69	15.56 ± 6.64	0.241	.810
BFM of right arm (kg)	1.09 ± 0.47	1.08 ± 0.61	0.082	.935
BFM of left arm (kg)	1.11 ± 0.47	1.09 ± 0.60	0.178	.859
BFM of right leg (kg)	2.73 ± 0.89	2.63 ± 0.99	0.474	.637
BFM of left leg (kg)	2.71 ± 0.88	2.63 ± 0.99	0.421	.675
BFM of trunk (kg)	7.31 ± 2.92	7.19 ± 3.38	0.176	.860
Fat-free mass indictors of BC				
FFM (kg)	40.59 ± 5.96	41.04 ± 6.10	-0.357	.722
SLM (kg)	38.09 ± 5.63	38.54 ± 5.74	-0.385	.701
SMM (kg)	6.32 ± 0.73	6.33 ± 0.70	-0.072	.943

BC = body composition, BEG = the Baduanjin exercise training group, BFM = body fat mass, BMI = body mass index, BMR = basal metabolic rate, CG = the control group, FFM = fat-free mass, PBF = percent body fat, SLM = soft lean mass, SMM = skeletal muscle mass, TBW = total body water, WHR = waist–hip ratio.

### 3.2. Comparison of body mass and body composition indicators between groups

Table 2 shows a comparison of the differences in body weight and body composition indicators between the 2 groups from 12 weeks to baseline. After 12 weeks of intervention, the differences in BMI and WHR in the body mass from 12 weeks to baseline in the BEG were significantly higher than those in the CG (*P* < .05) with a moderate effect size (Cohen d = 0.584 and 0.474). Linear mixed model analysis showed a significant interaction effect of group by time (*P* = .007 and .028).

**Table 2:** Comparison of the differences in body mass and body composition indicators between groups from 12 weeks to baseline.

Variables	BEG (MD ± SE)	CG (MD ± SE)	*P*-value	Cohen d	Time × group interaction F/*P*-value
Body mass					
BMI/(kg/m^2^)	-0.554 ± 0.093	-0.049 ± 0.177	.007	-0.584	7.600/.007
WHR (%)	-0.001 ± 0.002	0.0081 ± 0.004	.028	-0.474	5.003/.028
Overall indicators of BC					
BMR (kcal/d)	-0.554 ± 4.159	-7.622 ± 5.298	.293	0.224	1.117/.293
TBW (kg)	-0.043 ± 0.139	-0.276 ± 0.182	.305	0.219	1.065/.305
Total protein (kg)	0.002 ± 0.038	-0.027 ± 0.048	.639	0.100	0.222/.639
Total mineral (kg)	0.019 ± 0.019	-0.040 ± 0.255	.066	0.394	3.452/.066
Fat mass indicators of BC					
PBF (%)	-1.182 ± 0.253	0.276 ± 0.610	.014	-0.529	6.224/.014
BFM (kg)	-0.943 ± 0.167	0.211 ± 0.450	.007	-0.584	7.602/.007
BFM of right arm (kg)	-0.077 ± 0.017	-0.019 ± 0.035	.008	-0.578	7.438/.008
BFM of left arm (kg)	-0.075 ± 0.016	0.022 ± 0.037	.008	-0.577	7.429/.008
BFM of right leg (kg)	-0.152 ± 0.033	0.011 ± 0.075	.028	-0.472	4.965/.028
BFM of left leg (kg)	-0.154 ± 0.328	0.000 ± 0.073	.036	-0.452	4.554/.036
BFM of trunk (kg)	-0.470 ± 0.081	0.151 ± 0.229	.004	-0.624	8.669/.004
Fat free mass indicators of BC					
FFM (kg)	-0.027 ± 0.194	-0.354 ± 0.246	.296	0.223	1.106/.296
SLM (kg)	-0.039 ± 0.178	-0.343 ± 0.230	.295	0.223	1.110/.295
SMM (kg)	-0.077 ± 0.023	-0.092 ± 0.033	.699	0.259	0.151/.699

BC = body composition, BEG = the Baduanjin exercise training group, BFM = body fat mass, BMI = body mass index, BMR = basal metabolic rate, CG = the control group, FFM = fat free mass, MD = mean difference, PBF = percent body fat, SE = standard error, SLM = soft lean mass, SMM = skeletal muscle mass, TBW = total body water, WHR = waist–hip ratio.

For the total body composition indicators, the differences in the BMR, TBW, total protein, and total minerals from 12 weeks to baseline between the 2 groups were not found to be statistically significant. For BFM indicators, the differences in PBF, BFM, and BFM of both arms, both legs and trunk from 12 weeks to baseline in the BEG were significantly lower than those in the CG (all *P* < .05) with a moderate effect size (Cohen d from 0.452 to 0.624) and a significant interaction effect of group by time (all *P* < .05) through the mixed linear model analysis. FFM indicators, no statistical significance was found (all *P* > .05) in the differences of FFM, SLM, SMM indicators from 12 weeks to baseline between the 2 groups.

## 4. Discussion

In this randomized controlled trial of Baduanjin exercise on health-related fitness of college students, we found that compared with the CG without specific exercise intervention, 12 weeks of regular Baduanjin exercise training could significantly reduce the BMI and WHR indicator of body mass, and reduce PBF, BFM, and BFM of both arms, both legs, and trunk in fat mass indicators of body composition of college students, with a moderate effect size and a significant interaction effect of intervention by time, but no significant differences were found in FFM indicators such as FFM, SLM, and SMM. These findings suggest that regular Baduanjin exercise training may be effective in controlling body mass by reducing fat mass of body composition in college students.

It is well known that regular exercise training/physical activity is helpful for a healthier body composition, especially in individuals with obesity or overweight,^[27]^ therefore it is commonly recommended as an intervention to improve body composition.^[28]^ A growing number of studies have already demonstrated that regular Baduanjin training can unite the body and mind to positively influence physical function and psychological status.^[16,18]^ Several previous studies also reported that Baduanjin exercise training for more than 8 weeks could significantly reduce body weight, BMI, or waist circumference in overweight adults.^[29,30]^ The results of the current study showed that 12 weeks of regular Baduanjin exercise training significantly reduced the BMI and WHR in college students. These findings are consistent with previous studies that found that the body weight and BMI of healthy adults with normal BMI in the BEG were also significantly reduced after 16 weeks of Baduanjin intervention compared with no exercise intervention.^[31]^ But the current study did not find that the differences of total body composition indicators including BMR, TBW, total protein, and total minerals from the 12 weeks to baseline in the BEG were significantly higher than them in the CG, suggesting that regular Baduanjin exercise reduces body mass but may not change the BMR, TBW, total protein, and total minerals. Baduanjin exercise consists of 8 fluid movements and postures, each of which focuses on the function of one part of the body, and emphasizes gentle body and limb movements guided by a calm mind and spontaneous breathing to promote harmony between mind and body during practice.^[16]^ For example, its second movement is called “Drawing the Bow to Shoot the Eagle”; this movement requires the practitioner to imitate the action of drawing a bow to each side with a lower horse stance posture, focusing mainly on the waist area. In short, when practicing Baduanjin, practitioners must maintain a stable center of gravity and use the lumbar spine as the axis to drive the movement of the 4 limbs, while alternately changing the muscle tension and relaxation in different parts of the body.^[24]^ These characteristics of the Baduanjin exercise can provide a reasonable explanation for the improvement in the practitioner’s body mass.

Body composition generally consists of the relative components of fat mass and lean mass in the body, and these 2 components and their interaction have important implications for human health.^[32]^ It is believed that regular aerobic exercise training may be more beneficial in reducing BFM, while resistance exercise training may contribute to increasing body FFM and reducing body fat content.^[33]^ One study reported that 12 weeks of supervised aerobic exercise could significantly reduce body mass, BMI, and waist circumference of overweight women by reducing fat mass.^[34]^ As one of the traditional Chinese qigong exercises, Baduanjin exercise belongs to the type of low-to-moderate intensity aerobic exercise. Our results also showed that compared with the no exercise intervention CG, 12 weeks of Baduanjin exercise intervention significantly reduced the fat mass indicators of body composition including total PBF and BFM, and BFM in both arms, both legs and trunk with moderate effect size, while in the FFM indicators including FFM, SLM, and SMM, the decline changes from 12 weeks to baseline in the Baduanjin training group was lower than those in the CG, but no significant difference was found between the 2 groups, these results suggest the possible mechanism that 12 weeks of Baduanjin exercise intervention reduce the body mass mainly through reducing the fat mass of body composition. A recent systematic review found that regular aerobic exercise training can significantly improve the body composition in young and middle-aged adults, through improvements in PFM and fat mass, but not changes in FFM.^[35]^

Some strengths and limitations of this study should be noted. The first strength of this study is its design as a randomized controlled trial and strong quality controls; secondly, the study had high adherence to the assigned intervention;^[24]^ finally, body composition indicators were objectively assessed using the Inbody 720 instrument, which has higher validity and reliability than commercially available bioimpedance scales used to assess body composition.^[36]^ However, several limitations of this study should be considered. First, the sample size may not be large enough to meet the statistical power; second, too many participants in the CG who did not complete 2 Inbody tests (baseline and after 12 weeks) may also result in unanticipated bias.

## 5. Conclusion

The results of this study show that 12 weeks of Baduanjin exercise training can significantly reduce body mass and BFM in college students. Therefore, regular Baduanjin exercise training may be beneficial to improve body composition in young adults.

## Author contributions

**Conceptualization:** Guohua Zheng.

**Data curation:** Fang Zhao, Shanshan Sun.

**Formal analysis:** Nana Wen.

**Investigation:** Shanshan Sun, Jian Xiong.

**Project administration:** Guohua Zheng.

**Writing – original draft:** Nana Wen.

**Writing – review & editing:** Guohua Zheng.
